# Supplementary material for: Chemogenomic profiling to understand the antifungal action of a bioactive aurone compound
Source: PLoS One. 2019 Dec 11;14(12):e0226068. doi: 10.1371/journal.pone.0226068 (PMC6905557; doi:10.1371/journal.pone.0226068)
Supplement: S1 Table — (DOCX) [file pone.0226068.s001.docx]

**S1 Table:** Strains used in this study.

| **Strains** | **Relevant characteristics or genotype** | **Reference** |
| --- | --- | --- |
| *Candida albicans* ATCC 90028 | Reference strain | [1] |
| *C. albicans* ATCC 90029 | Reference strain | [1] |
| *C. albicans*M1: SC5314 | Wild-type *C. albicans* model strain | [2] |
| *C. glabrata* ATCC 66032 | Reference strain | [1] |
| *C. tropicalis* ATCC 750 | Reference strain | [1] |
| *C. albicans*M4: Gu2 | Clinical isolate from patient G, fluconazole-susceptible | [3] |
| *C. albicans*M6: F1 | Clinical isolate from patient F, fluconazole-susceptible | [3] |
| *C. albicans* M5: Gu5 | Clinical isolate from patient G, fluconazole-resistant, gain of function mutation in TF *TAC1* | [3] |
| *C. albicans* M7: F5 | Clinical isolate from patient F, fluconazole-resistant, gain of function mutation in TF *MRR1* | [3] |
| *C. albicans* M2: ScTAC1R34A | *TAC1*^G980E^ mutation, fluconazole-resistant | [4] |
| *C. albicans* M3: ScMRR1R34A | *MRR1^P683S^* mutation, fluconazole-resistant | [5] |
| *C. albicans*ATCC 64124 | Multidrug resistant isolate, resistant to amphotericin B, caspofungin, fluconazole, and 5-fluorocytosine except and high concentrations | [6] |
| *Saccharomyces cerevisiae* S288C | Grandparent strain to chemogenomic mutants |  |

**References:**

1. Institute, C.a.L.S., *Reference method for broth dilution antifungal susceptibility testing of yeasts.* Approved Standard-Third Edition M27-A3, 2008. **28**.

2. Gillum, A.M., E.Y.H. Tsay, and D.R. Kirsch, *Isolation of the Candida albicans gene for orotidine-5′-phosphate decarboxylase by complementation of S. cerevisiae ura3 and E. coli pyrF mutations.* Molecular and General Genetics MGG, 1984. **198**(1): p. 179-182.

3. Franz, R., et al., *Multiple molecular mechanisms contribute to a stepwise development of fluconazole resistance in clinical Candida albicans strains.* Antimicrob Agents Chemother, 1998. **42**(12): p. 3065-72.

4. Sasse, C., et al., *The transcription factor Ndt80 does not contribute to Mrr1-, Tac1-, and Upc2-mediated fluconazole resistance in Candida albicans.* PloS one, 2011. **6**(9): p. e25623-e25623.

5. Schubert, S., et al., *Regulation of efflux pump expression and drug resistance by the transcription factors Mrr1, Upc2, and Cap1 in Candida albicans.* Antimicrobial agents and chemotherapy, 2011. **55**(5): p. 2212-2223.

6. Ryley, J.F., R.G. Wilson, and K.J. Barrett-Bee, *Azole resistance in Candida albicans.* Sabouraudia, 1984. **22**(1): p. 53-63.
