# Supplementary material for: Chemogenomic profiling to understand the antifungal action of a bioactive aurone compound
Source: PLoS One. 2019 Dec 11;14(12):e0226068. doi: 10.1371/journal.pone.0226068 (PMC6905557; doi:10.1371/journal.pone.0226068)
Supplement: S4 Table — (DOCX) [file pone.0226068.s004.docx]

**S4 Table:** Enrichment analysis of KEGG pathway and gene ontology (biological process, molecular function, and cellular component) of deleted-gene names of 238 significantly (FDR < 0.05 and fold change ≥ 1.5) responsive heterozygous (HIP-profile) and homozygous (HOP-profile) mutants to aurone SH1009. Hypergeometric testing through ClueGo app was used to find the significantly enriched KEGG/GO terms using GO categories in the *Saccharomyces cerevisiae*-S288C as a background with FRD < 0.05 as a cutoff significant value:

| **Ontology Source** | **GO-Term** | ***P-*value** | **No. of Genes** | **Associated Genes from HOP Profile** | **Associated Genes from HIP Profile** |
| --- | --- | --- | --- | --- | --- |
| **KEGG Pathway** | Cell cycle | 0.0000267 | 15 | BUB1, CLB3, CLN2, FOB1, LTE1, SLK19, SWI4 | APC1, APC4, CDC28, CDC5, MCM2, MCM7, MOB1, ORC4 |
|  | Meiosis | 0.000536 | 13 | BUB1, CLB3, CLN2, SLK19, SWI4, UME6 | APC1, APC4, CDC28, CDC5, MCM2, MCM7, ORC4 |
|  | Ribosome | 0.299472 | 9 | RPL27A, RPL41A, RPL4A, RPS1B, RPS24B, RPS30B, RPS4A | RPL3, RPS31 |
|  | Endocytosis | 0.01292 | 7 | CHM7, EDE1, GLO3 | ARC15, CDC42, RHO1, RSP5 |
|  | DNA replication | 0.003922 | 5 |  | MCM2, MCM7, POL3, RFC1, RFC3 |
|  | Nicotinate and nicotinamide metabolism | 0.004427 | 4 | HST2, HST4, ISN1, SDT1 |  |
| **Biological Process** | Cell division | 0.0000726 | 25 | BOI2, CLB3, CLN2, EDE1, ELM1, END3, LTE1, SLK19, TOF2, VPS51 | APC1, APC4, CBK1, CDC13, CDC25, CDC28, CDC34, CDC42, CDC5, GNA1, MCM7, MOB1, RFC1, RHO1, SPC24 |
|  | Exit from mitosis | 0.000155 | 8 | GIC1, GIC2, LTE1, TOF2 | APC1, CDC42, CDC5, MOB1 |
|  | cell budding | 0.001004 | 8 | BOI2, ELM1, END3, VPS51 | CBK1, CDC28, CDC42, RHO1 |
|  | DNA replication | 0.001418 | 14 | CSM3, FOB1, TOF1 | CDC28, CDC34, IPI1, MCM2, MCM7, ORC4, POL3, PSF3, RFC1, RFC3, SLD2 |
|  | Cytoskeleton organization | 0.002668 | 19 | ARC15, ARK1, CBK1, CDC28, CDC42, CDC5, CLB3, ELM1, END3, GIC1, GIC2, KIP3, MPS3, NIP100, RHO1, RSP5, SLK19, SPC24, TSC11 | ARC15, CBK1, CDC28, CDC42, CDC5, MPS3, RHO1, RSP5, SPC24, TSC11 |
|  | Regulation of endocytosis | 0.002952 | 4 | ARK1, LDB19 | RHO1, RSP5 |
|  | Actin cortical patch organization | 0.003639 | 4 | ARK1, EDE1, END3 | ARC15 |
|  | Establishment of cell polarity | 0.003903 | 8 | BOI2, END3, GIC1, GIC2, KIP3, NIP100 | CDC42, TSC11 |
|  | Septin ring organization | 0.003922 | 5 | ELM1, GIC1, GIC2 | CDC42, RHO1 |
|  | Regulation of cellular carbohydrate metabolic process | 0.017427 | 5 | FLO8, PCL6, UME6 | CDC28, RHO1 |
|  | tRNA processing | 0.005963 | 11 | ELP2, IKI3, MSF1, POA1, PTC1, TGS1, TRM13 | CEG1, CLP1, PTA1, RSP5 |
|  | Receptor-mediated endocytosis | 0.00721 | 3 | LDB19 | RSP5, YPP1 |
|  | ncRNA transcription | 0.017205 | 6 | HHT1 | NCB2, RPA135, RPC17, RRN3, RRN9 |
|  | DNA repair | 0.021107 | 18 | BLM10, CSM3, EAF5, HHT1, IES2, MLH3, RAD16, RAD18, TOF1 | CDC28, MCM2, MCM7, POL3, PSF3, RFC1, RFC3, SLD2, SPP382 |
|  | Chromatin silencing | 0.025447 | 11 | FOB1, GIC1, HST2, HST4, SCS2, SPT21, TOF2, YAP5 | MCM7, MPS3, ORC4 |
|  | Cell morphogenesis | 0.026637 | 5 | ELM1, GIC1, GIC2 | CDC42, RSP5 |
|  | Phosphorylation | 0.05002 | 22 | ACK1, ARK1, BUB1, CLB3, CLN2, COX9, DAS2, ELM1, MOB1, PCL6, PKP1, PRR1, PTC1, RTK1, TOS3, YJL045W, YPK3 | CBK1, CDC28, CDC5, CLP1, TSC11 |
|  | Vesicle-mediated transport | 0.05002 | 22 | APS1, ARK1, BOI2, EDE1, ELO3, EMP46, END3, FEN2, GGA2, GLO3, LDB19, LTE1, PEP12, VAM7, VPS51, YPP1 | CDC28, CDC42, RHO1, RSP5, SEC12, SEC24, YPP1 |
| **Molecular Function** | Nucleotide binding | 0.000382 | 51 | AIM10, ARK1, BUB1, CUS2, DAS2, ELM1, ELO3, ERV1, GSP2, GTF1, HST2, HST4, KIP3, MEF2, MLH3, MSD1, MSF1, PKP1, PRR1, RAD16, RSP5, RTK1, SRP101, TOS3, YJL045W, YPK3, ZIP2 | CBK1, CDC28, CDC34, CDC42, CDC5, CEG1, CLP1, DED81, ERV1, GPN2, LSG1, MCM2, MCM7, MGE1, MPS3, PGA3, POL3, RFC1, RFC3, RHO1, RPC17, RSP5, TFG2, YDR341C, YHR020W |
|  | Aminoacyl-tRNA ligase activity | 0.002047 | 6 | AIM10, MSD1, MSF1 | DED81, YDR341C, YHR020W |
|  | Ribonucleoside binding | 0.003384 | 40 | AIM10, ARK1, BUB1, DAS2, ELM1, ELO3, GSP2, GTF1, KIP3, MEF2, MLH3, MSD1, MSF1, PKP1, PRR1, RAD16, RTK1, SRP101, TOS3, YPK3 | CBK1, CDC28, CDC34, CDC42, CDC5, CEG1, CLP1, DED81, GPN2, LSG1, MCM2, MCM7, MPS3, RFC1, RFC3, RHO1, RPA135, TFG2, YDR341C, YHR020W |
|  | DNA binding | 0.003933 | 33 | CRP1, CSM3, FLO8, FOB1, HHT1, IES2, IOC2, LRP1, MLH3, RAD16, RAD18, STP2, SWI4, TOF2, UME6, VID22, YAP5, ZIP2 | CDC13, CDC5, MCM2, MCM7, NCB2, ORC4, POL3, RFC1, RFC3, RPA135, RRN3, RRP14, SLD2, TAF9, TFG2 |
|  | Protein kinase activity | 0.015 | 14 | ACK1, ARK1, BUB1, DAS2, ELM1, PKP1, PRR1, RTK1, TOS3, YPK3 | CBK1, CDC28, CDC5, CLP1 |
|  | GTP binding | 0.024937 | 10 | CEG1, GSP2, MEF2, GIC1, GIC2 | CDC42, GPN2, LSG1, RHO1, SRP101 |
|  | ATP binding | 0.049726 | 31 | AIM10, ARK1, BUB1, DAS2, ELM1, ELO3, KIP3, MPS3, MSD1, MSF1, PKP1, PRR1, RAD16, RTK1, TFG2, TOS3, YDR341C, YPK3 | CBK1, CDC28, CDC34, CDC5, CLP1, DED81, MCM2, MCM7, MPS3, RFC1, RFC3, TFG2, YDR341C, YHR020W |
| **Cellular Component** | Nucleus | 0.0000013 | 105 | APS1, BLM10, BUB1, CLB3, CLN2, CMG1, CRP1, CSM3, CUS2, DAS2, EAF5, ELO3, ELP2, FLO8, FOB1, FYV7, GSP2, HHT1, HST2, HST4, ICP55, IES1, IES2, IKI3, IOC2, IPI1, KIP3, LIN1, LRP1, MLH3, PCL6, PTC1, RAD16, RAD18, RBD2, RPL4A, RPS31, RSF1, RTK1, SCS2, SHE2, SKI8, SLK19, STP2, SVF1, SWI4, TGS1, TOF1, TOF2, TOS3, TRM13, UBP2, UME6, VID22, YAP5, YOR131C, YPK3, ZIP2 | APC1, APC4, CBK1, CDC13, CDC25, CDC28, CDC34, CDC42, CDC5, CEG1, CLP1, FAF1, FCF1, GNA1, IPI1, MCM2, MCM7, MED7, MOB1, MPS3, NCB2, NOC2, NOP14, NUT2, ORC4, POL3, PRE1, PSF3, PTA1, RDS3, RFC1, RFC3, RPA135, RPC17, RPF2, RPS31, RRN3, RRN9, RRP1, RRP14, RSP5, SLD2, SPC24, SPN1, SPP382,TAF5, TAF9, TFG2, YDR341C |
|  | Replication fork | 0.000224 | 9 | CSM3, HHT1, TOF1 | MCM2, MCM7, POL3, PSF3, RFC1, RFC3 |
|  | Cytoskeleton | 0.000981 | 19 | ARK1, BOI2, EDE1, ELM1, END3, GIC1, GIC2, KIP3, MOB1, NIP100, SLK19 | APC1, ARC15, CDC28, CDC42, CDC5, MPS3, RSP5, YPP1 |
|  | Cellular bud | 0.002811 | 16 | BOI2, EDE1, ELM1, GIC1, GIC2, LTE1, MOB1, SCS2, SHE2, YNL058C | CBK1, CDC28, CDC42, CDC5, RHO1, RSP5 |
|  | Cellular bud tip | 0.003287 | 9 | EDE1, GIC1, GIC2, SCS2, SHE2 | CBK1, CDC42, RHO1, RSP5 |
|  | Cellular bud neck | 0.007523 | 13 | BOI2, EDE1, ELM1, GIC1, GIC2, MOB1, SCS2, YNL058C | CBK1, CDC28, CDC42, CDC5, RHO1 |
|  | Cytoskeletal part | 0.008832 | 16 | EDE1, ELM1, END3, KIP3, MOB1, NIP100, SLK19 | APC1, ARC15, ARK1, CDC28, CDC42, CDC5,MPS3, RSP5, YPP1 |
|  | Site of polarized growth | 0.011329 | 15 | BOI2, EDE1, ELM1, GIC1, GIC2, MOB1, SCS2, SHE2, YNL058C | CBK1, CDC28, CDC42, CDC5, RHO1, RSP5 |
|  | actin cortical patch | 0.017205 | 6 | ARK1, EDE1, END3 | ARC15, RSP5, YPP1 |
|  | nuclear pre-replicative complex | 0.019613 | 3 |  | MCM2, MCM7, ORC4 |
|  | Actin cytoskeleton | 0.026133 | 7 | ARK1, EDE1, END3, NIP100 | ARC15, RSP5, YPP1 |
|  | Golgi apparatus | 0.046058 | 16 | APS1, BOI2, EMP46, GGA2, GLO3, LDB19, MNN2, PEP12, RBD2, VPS51, YCR007C | RHO1, RSP5, PIS1, SEC12, SEC24 |
