# Supplementary material for: Chemogenomic profiling to understand the antifungal action of a bioactive aurone compound
Source: PLoS One. 2019 Dec 11;14(12):e0226068. doi: 10.1371/journal.pone.0226068 (PMC6905557; doi:10.1371/journal.pone.0226068)
Supplement: S5 Table — (DOCX) [file pone.0226068.s005.docx]

**S5 Table:** A set of 24 forward indexed primers containing (Illumina-specific region, 10 bp index tag [highlighted], and 18 bp common priming site U1). The common reverse primer contains (Illumina-specific region and a common priming site from the resistant KanMX gene region) [1].

| **Name of Primer** | **Sequence (5' - 3')** |
| --- | --- |
| Non_Essentail_1009_1 | AATGATACGGCGACCACCGAGATCTACACTCTTTCCCTACACGACGCTCTTCCGATCTAATAGGCGCTGATGTCCACGAGGTCTCT |
| Non_Essentail_1009_2 | AATGATACGGCGACCACCGAGATCTACACTCTTTCCCTACACGACGCTCTTCCGATCTTACAGTTGCGGATGTCCACGAGGTCTCT |
| Non_Essentail_1009_3 | AATGATACGGCGACCACCGAGATCTACACTCTTTCCCTACACGACGCTCTTCCGATCTATCCTAGCAGGATGTCCACGAGGTCTCT |
| Non_Essentail_1009_4 | AATGATACGGCGACCACCGAGATCTACACTCTTTCCCTACACGACGCTCTTCCGATCTGATTAGCCTCGATGTCCACGAGGTCTCT |
| Non_Essentail_MMS_1 | AATGATACGGCGACCACCGAGATCTACACTCTTTCCCTACACGACGCTCTTCCGATCTAATGAGCCGTGATGTCCACGAGGTCTCT |
| Non_Essentail_MMS_2 | AATGATACGGCGACCACCGAGATCTACACTCTTTCCCTACACGACGCTCTTCCGATCTACGCGGATTAGATGTCCACGAGGTCTCT |
| Non_Essentail_MMS_3 | AATGATACGGCGACCACCGAGATCTACACTCTTTCCCTACACGACGCTCTTCCGATCTGCTTACGGAAGATGTCCACGAGGTCTCT |
| Non_Essentail_MMS_4 | AATGATACGGCGACCACCGAGATCTACACTCTTTCCCTACACGACGCTCTTCCGATCTCGGTAGACTAGATGTCCACGAGGTCTCT |
| Non_Essentail_DMSO_1 | AATGATACGGCGACCACCGAGATCTACACTCTTTCCCTACACGACGCTCTTCCGATCTATTGCCGGAAGATGTCCACGAGGTCTCT |
| Non_Essentail_DMSO_2 | AATGATACGGCGACCACCGAGATCTACACTCTTTCCCTACACGACGCTCTTCCGATCTGACATGCTAGGATGTCCACGAGGTCTCT |
| Non_Essentail_DMSO_3 | AATGATACGGCGACCACCGAGATCTACACTCTTTCCCTACACGACGCTCTTCCGATCTTACGCTGCATGATGTCCACGAGGTCTCT |
| Non_Essentail_DMSO_4 | AATGATACGGCGACCACCGAGATCTACACTCTTTCCCTACACGACGCTCTTCCGATCTGTCAAGCACTGATGTCCACGAGGTCTCT |
| Essentail_1009_1 | AATGATACGGCGACCACCGAGATCTACACTCTTTCCCTACACGACGCTCTTCCGATCTAGCGTATGTCGATGTCCACGAGGTCTCT |
| Essentail_1009_2 | AATGATACGGCGACCACCGAGATCTACACTCTTTCCCTACACGACGCTCTTCCGATCTGCGGATTAACGATGTCCACGAGGTCTCT |
| Essentail_1009_3 | AATGATACGGCGACCACCGAGATCTACACTCTTTCCCTACACGACGCTCTTCCGATCTATACCTCGGAGATGTCCACGAGGTCTCT |
| Essentail_1009_4 | AATGATACGGCGACCACCGAGATCTACACTCTTTCCCTACACGACGCTCTTCCGATCTGGTAGACATCGATGTCCACGAGGTCTCT |
| Essentail_MMS_1 | AATGATACGGCGACCACCGAGATCTACACTCTTTCCCTACACGACGCTCTTCCGATCTAGGTACCTACGATGTCCACGAGGTCTCT |
| Essentail_MMS_2 | AATGATACGGCGACCACCGAGATCTACACTCTTTCCCTACACGACGCTCTTCCGATCTCGATAACGCTGATGTCCACGAGGTCTCT |
| Essentail_MMS_3 | AATGATACGGCGACCACCGAGATCTACACTCTTTCCCTACACGACGCTCTTCCGATCTTACCGGAATGGATGTCCACGAGGTCTCT |
| Essentail_MMS_4 | AATGATACGGCGACCACCGAGATCTACACTCTTTCCCTACACGACGCTCTTCCGATCTTAGGACCAGTGATGTCCACGAGGTCTCT |
| Essentail_DMSO_1 | AATGATACGGCGACCACCGAGATCTACACTCTTTCCCTACACGACGCTCTTCCGATCTCTGCAAGTTCGATGTCCACGAGGTCTCT |
| Essentail_DMSO_2 | AATGATACGGCGACCACCGAGATCTACACTCTTTCCCTACACGACGCTCTTCCGATCTTCGCGATTCAGATGTCCACGAGGTCTCT |
| Essentail_DMSO_3 | AATGATACGGCGACCACCGAGATCTACACTCTTTCCCTACACGACGCTCTTCCGATCTGGTAACGTACGATGTCCACGAGGTCTCT |
| Essentail_DMSO_4 | AATGATACGGCGACCACCGAGATCTACACTCTTTCCCTACACGACGCTCTTCCGATCTAAGACCTGTGGATGTCCACGAGGTCTCT |
| Common reverse primer | CAAGCAGAAGACGGCATACGAGCTCTTCCGATCTGCACGTCAAGACTGTCAAGG |

**A**

**B**

**References:**

1. Piotrowski, J.S., et al., *Chemical Genomic Profiling via Barcode Sequencing to Predict Compound Mode of Action.* Methods Mol. Biol. (N. Y., NY, U. S.), 2015. **1263**(Chemical Biology): p. 299-318.
