# Supplementary material for: Chemogenomic profiling to understand the antifungal action of a bioactive aurone compound
Source: PLoS One. 2019 Dec 11;14(12):e0226068. doi: 10.1371/journal.pone.0226068 (PMC6905557; doi:10.1371/journal.pone.0226068)
Supplement: S6 Table — (DOCX) [file pone.0226068.s006.docx]

**S7 Table:** List of primers used for RT–PCR.

| **Gene Name** | **Oligonucleotide Primer Sequence (5'->3')** | **References** |
| --- | --- | --- |
| **CDC28** | F:GTTATCTGATTATCAACGTCAAGAAAA | [8] |
|  | R: TCTAATGCTTTATAAACAACCCCATA |  |
| **HGC1** | F: AATATGCAACCACCACCACC | [9] |
|  | R: GAAACAGCACGAGAACCAGC |  |
| **CDC25** | F: GGTGGTTGACGTTTGCTCAC | This study |
|  | R: GGGCAGGAGGCTTGGTATTT |  |
| **RAS1** | F: CAACTATTGAGGATTCTTATCG | [10] |
|  | R: CGGTTCTCATATATTGTTCTC |  |
| **RAS2** | F: CAATCATTCACTGCATTAGAAG | [10] |
|  | R: CAAATTCTGCTCCTTCATAATAG |  |
| **CDC42** | F: GGGTGAAAAATTGGCTAAGGAA | [11] |
|  | R: CCTCTTTGAGTCAATGCAGAACA |  |
| **MCM2** | F: CATCAAGAAGTTCACGTTAG | [12] |
|  | R: CAGTATTCGAATCTTGAACG |  |
| **TCS11** | F: ATCCCATCACGCAGCATTGA | This study |
|  | R: TCGGCGGGCAAATAGTTGTT |  |
| **RPS4A** | F:TGCTTACTTATTGTTAGTTCAAGGTGGTA | [13] |
|  | R: CAACACCAACGGATTCCAATAAA |  |
| **RSP5** | F: GGTTGGGAACAAAGATTTAC | [14] |
|  | R: GAGCAGTATTGGTTAATCTC |  |
| **CBK1** | F: CCGCAAATGTCGGCATTCAT | This study |
|  | R: TCGATGCTGGTGGTTGGAAA |  |
| **GAPDH** | F: CGGTCCATCCCACAAGGA | [15] |
|  | R: AGTGGAAGATGGGATAATGTTACCA |  |

**References:**

8. Mitra, S., et al., *Rad51-Rad52 mediated maintenance of centromeric chromatin in Candida albicans.* PLoS Genet, 2014. **10**(4): p. e1004344.

9. Greig, J.A., et al., *Cell cycle-independent phospho-regulation of Fkh2 during hyphal growth regulates Candida albicans pathogenesis.* PLoS Pathog, 2015. **11**(1): p. e1004630.

10. Zhu, Y., et al., *Ras1 and Ras2 play antagonistic roles in regulating cellular cAMP level, stationary-phase entry and stress response in Candida albicans.* Mol Microbiol, 2009. **74**(4): p. 862-75.

11. Bassilana, M., J. Hopkins, and R.A. Arkowitz, *Regulation of the Cdc42/Cdc24 GTPase module during Candida albicans hyphal growth.* Eukaryot Cell, 2005. **4**(3): p. 588-603.

12. Xie, J.L., et al., *The Candida albicans transcription factor Cas5 couples stress responses, drug resistance and cell cycle regulation.* Nat Commun, 2017. **8**(1): p. 499.

13. Lu, H., et al., *Loss of RPS41 but not its paralog RPS42 results in altered growth, filamentation and transcriptome changes in Candida albicans.* Fungal Genet Biol, 2015. **80**: p. 31-42.

14. Leach, M.D. and L.E. Cowen, *Membrane fluidity and temperature sensing are coupled via circuitry comprised of Ole1, Rsp5, and Hsf1 in Candida albicans.* Eukaryot Cell, 2014. **13**(8): p. 1077-84.

15. Nailis, H., et al., *Development and evaluation of different normalization strategies for gene expression studies in Candida albicans biofilms by real-time PCR.* BMC Mol Biol, 2006. **7**: p. 25.
