# Supplementary figures and images for: Chemogenomic profiling to understand the antifungal action of a bioactive aurone compound
Source: PLoS One. 2019 Dec 11;14(12):e0226068. doi: 10.1371/journal.pone.0226068 (PMC6905557; doi:10.1371/journal.pone.0226068)

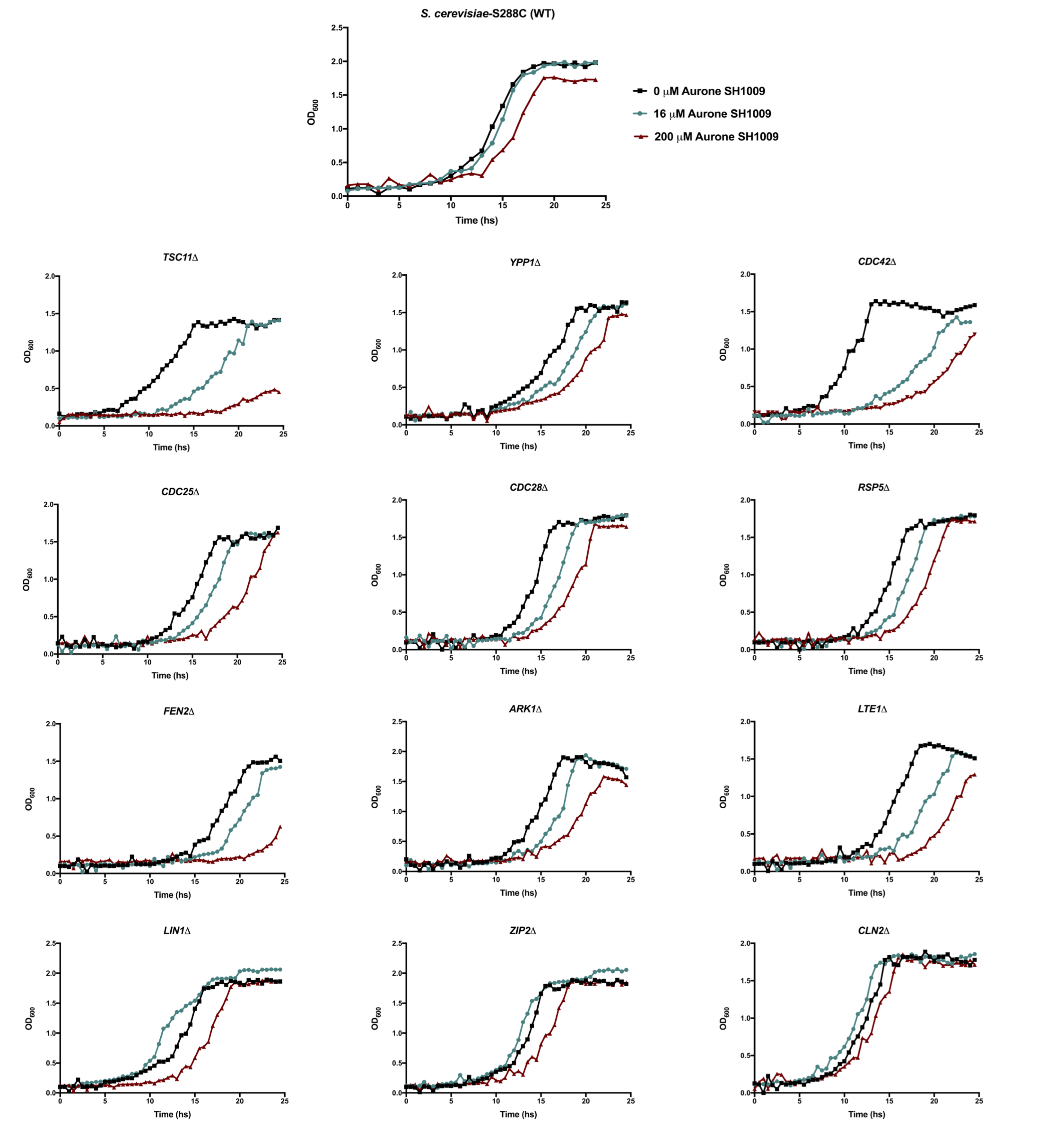

Supplement: S1 Fig — (TIF) [file pone.0226068.s007.tif]
