# Supplementary material for: Chemogenomic profiling to understand the antifungal action of a bioactive aurone compound
Source: PLoS One. 2019 Dec 11;14(12):e0226068. doi: 10.1371/journal.pone.0226068 (PMC6905557; doi:10.1371/journal.pone.0226068)
Supplement: S1 File — (DOCX) [file pone.0226068.s008.docx]

Synthesis of SH1009 [ (*Z*)-6,7-dihydroxy-2-(3-hydroxy-4-methoxybenzylidene)benzofuran-3(2*H*)-one ]

To a solution of 6,7-dihydroxybenzofuranone (88 mg, 0.5 mmol) and 3-hydroxy-4-methoxybenzaldehyde (78 mg, 0.51 mmol) in glacial acetic acid (3 mL) was added 3 drops of concentrated hydrochloric acid. The mixture was stirred for 3 hours at room temperature. The mixture was diluted with deionized water (6 mL) to form the product as a tan precipitate (45 mg) that was separated by filtration and washed with an additional 2 mL of water. The aqueous layer retained significant amounts of product. Repeated extraction with ethyl acetate (3 x 10 mL), followed by evaporation of the solvent and extracted acetic acid, followed by trituration with diethyl ether (10 mL) afforded an additional 20 mg of the product. Total yield of product was 65 mg (44% yield), with spectral and physical properties consistent with those reported in the literature. Tan solid (MP = 275-280 °C). IR (neat, thin film): 3100-3500, 1700, 1400, 1200 cm^-1^; ^1^H NMR (DMSO, 300 MHz) 10.78 (s, 1H), 9.54 (s, 1H), 9.27 (s, 1H), 7.53 (d, J = 2.1 Hz, 1H), 7. 42 (dd, J_1,3_ = 8.4 Hz, J_1,2_ = 2.1 Hz, 1H), 7.09 (d, J = 8.1 Hz, 1H), 7.01 (d, J = 8.4 Hz, 1H), 6.75 (d, J = 8.4 Hz, 1H), 6.62 (s, 1H), 3.81 (s, 3H); ^13^C NMR (DMSO, 75 MHz) 182.64, 155.59, 154.92, 150.00, 147.09, 146.84, 130.68, 125.42, 124.73, 118.28, 115.80, 114.91, 113.296, 112.62, 111.74.
